# Practices and knowledge of female gynecologists regarding contraceptive use: a real-world Chinese survey

**DOI:** 10.1186/s12978-018-0557-9

**Published:** 2018-06-26

**Authors:** Xin Yang, Xiaodong Li, Yanjie Wang, Xiaojing He, Yang Zhao

**Affiliations:** 1 Department of Obstetrics and Gynecology, Peking University People’s Hospital, Xicheng District, Beijing, 100044 China; 2 Department of Obstetrics and Gynecology, The Second Hospital of Hebei Medical University, Xinhua District, Beijing, Hebei Province China

**Keywords:** Contraception, Knowledge and personal choice, Female obstetricians/gynecologists, Combined oral contraceptives

## Abstract

**Background:**

There is no evidence of the practices of obstetricians and gynecologists (OB/GYNs) regarding contraceptive use and determinants influencing contraceptive choices, including emergency methods such as combined oral contraceptives (COCs) and levonorgestrel intrauterine system (LNGIUS). This survey determines the practices and knowledge among Chinese female OB/GYNs regarding modern contraceptive methods.

**Methods:**

A multicenter questionnaire was completed by 2000 female OB/GYNs participating in training courses organized by the gynecological endocrinology training committee of the Chinese Medical Doctor Association from February to May 2013.

**Results:**

This survey achieved a response rate of 51.4%. The rate of induced abortion among this group was 56.3%; this may be attributable to unreliable contraceptive methods (55.5%) and failure of reliable contraceptive methods (18.9%). Intrauterine devices (IUDs) were more commonly used by parous women than nulliparous women (42.6% vs 1.7%; *p* < 0.0001), followed by condom and rhythm method (24.2% vs 20.8%). However, nulliparous women commonly used rhythm method (48.3% vs 3.3%; *p* < 0.0001) and condoms (19.2% vs 10.5%; *p* = 0.008). OB/GYNs demonstrated misconceptions of OB/GYNs about COCs, such as the risk of breast cancer, amenorrhea and premature ovarian failure, and decreased fertility as reported by 37.10, 10.6, and 7.5% of the respondents, respectively.

**Conclusions:**

IUDs were commonly used by parous Chinese OB/GYNs. Unreliable contraceptive methods and misconceptions about the side effects of COCs may result in the high rate of unintended pregnancies. Hence, awareness of safe and effective contraceptive methods should be strengthened among OB/GYNs in China.

## Plain text summary

Obstetricians and gynecologists (OB/GYNs) play a major role in counselling and guiding patients on contraceptive use. They should be well aware of the benefits and risks of different contraceptives. To date, there is no evidence on the knowledge and practices of OB/GYNs regarding contraceptive use and factors influencing their contraceptive choices.

The current study was designed to determine the knowledge and contraceptive practices among OB/GYNs of reproductive age. 51.4% of 2000 of OB/GYNs responded to the survey. The rate of abortion was found to be 56.3%, which was reportedly due to unreliable contraceptive methods (55.5%) and failure of reliable contraceptive methods (18.9%). Intrauterine devices (IUDs) were more commonly used by parous women (42.6% vs 1.7%; *p* < 0.0001) followed by Condoms and the rhythm method (24.2% vs 20.8%) compared to nulliparous women. However, women without children more commonly used rhythm method (48.3% vs 3.3%; *p* < 0.0001) and condoms (19.2% vs 10.5%; *p* = 0.008) compared to parous women. Hence, OB/GYNs in China should be educated on the safe and effective use of contraceptive methods to guide the general population in choosing the appropriate contraceptive methods.

## Background

Contraceptive use is largely influenced by factors such as access to medical care, social networks, and healthcare providers. OB/GYNs can influence women’s choice of contraceptive [1]. The knowledge of OB/GYNs is influential in most women’s choice of conceptive methods, as well as in public policies and designing family planning programs [2, 3]. A previous multinational survey comprising 1001 healthcare professionals (HCPs) reported that the personal choice of contraceptives significantly influenced their recommendations to women [4]. However, it is evident from the literature that there are several misconceptions among HCPs that contribute to underuse of contraceptives. A study assessing family physicians’ perceptions of IUDs revealed that the majority of family physicians believed that pelvic inflammatory disease, ectopic pregnancies, and failure of IUDs were major risks and as a result did not prescribe IUDs [5]. Another study emphasized the need for education and training HCPs about the guidelines for contraceptive use to improve their knowledge and overcome misconceptions [6]. As China accounts for one-fifth of all abortions, there is an unmet need for contraceptive use in avoiding unplanned pregnancies and induced abortions [7]. Thus, the physician’s choice may help women select a contraceptive method that best suits their needs and lifestyles, thereby maximizing compliance and helping prevent unintended pregnancies [8]. In addition, China lags behind the rest of the world in the use of oral contraceptives, showing a downward trend in the past two decades from 4.44 to 0.98% [9]. A pivotal factor impeding women’s access to efficient contraceptives may be lack of awareness among Chinese OB/GYNs or the fear of unwanted side effects. Hence, understanding the knowledge and preferences of Chinese OB/GYNs about contraceptive methods is warranted. To date, there is no study on the knowledge of OB/GYNs regarding contraceptive use and determinants influencing contraceptive choices, including emergency methods such as combined oral contraceptives (COCs) and levonorgestrel intrauterine system (LNGIUS). The current study was designed to assess the knowledge and factors influencing the use of contraceptive methods among parous and nulliparous Chinese OB/GYNs.

## Methods

### Study design and participants

This multicenter questionnaire was developed to understand the contraceptive choice of Chinese female OB/GYNs and their practices on the use of general COCs and LNGIUS used worldwide. Female OB/GYNs participating in training courses on gynecological endocrinology organized by the gynecological endocrinology training committee of the Chinese Medical Doctor Association in multiple regions of China from February to May 2013 participated in the survey with voluntary consent.

The questionnaire was developed at the Peking University People’s Hospital, designed to obtain information, including age, height, weight, marital status, cities or areas they are from, grade of hospital and specialty, health conditions, parity, menstruation status, clinically confirmed chronic diseases, choice of contraception, abortion because of unintended pregnancy, and their knowledge about COCs and LNGIUS. The participants were administered paper-based questionnaire. The survey was approved by the Clinical Research Ethics Committee of the Peking University People’s Hospital.

### Statistical methods

Data were analyzed using SPSS Statistics version 18.0 (Chicago: SPSS Inc. IBM Corp). Implausible answers and coding errors were carefully reviewed and corrected. The results are presented as counts and percentages. Chi square and ‘t’ test was used to compare categorical and continuous data, respectively. A probability value of < 0.05 was considered to be statistically significant.

## Results

### Demographic characteristics of participants

A total of 2000 questionnaires were distributed. The response rate was 51.4% (1027/2000). All the respondents were involved in clinical work related to contraception, birth control, and/or induced abortion in Class-I (5.8%), Class-II (42%), and Class III (52.2%) hospitals. Majority of the respondents were middle-aged (mean age: 40.0 ± 9.2 years). Of the 1027 respondents, 12 (1.2%) were unmarried and sexually inactive, and the remaining 1015 (98.8%) were married and sexually active. Majority of the respondents were parous (83.6%, 859/1027). A total of 197 OB/GYNs had amenorrhea (menopause or total hysterectomy). Of the remaining 830 OB/GYNs, 692 (83.4%) reported regular menstrual cycles and 138 (16.6%) reported irregular cycles. Among the 1015 female OB/GYNs who were sexually active, 571 (56.3%) had undergone induced abortion because of unintended pregnancy. The reported rate of unintended pregnancies due to an unreliable contraceptive method (rhythm method and lactation amenorrhea) was high at 55.5%. Other causes for unintended pregnancies were reported failure of reliable contraceptive methods, such as condom slipping or breakage, missing of COCs, and IUD falling out or moving down (18.9%). Further demographic characteristics of the respondents are given in Table 1.

**Table 1:** Demographic characteristics of the respondents

Characteristics	Number of respondents, *n* (%) *N* = 1027
Age in years	
22–29	126 (12.3)
30–39	359 (35.0)
40–49	355 (34.6)
50–59	137 (13.3)
≥ 60	50 (4.9)
Marital status	
Married/cohabiting	1015 (98.8)
Single	12 (1.2)
Parity	
Parous	859 (83.6)
Nulliparous	168 (16.4)
Grade of hospital	
1	60 (5.8)
2	431 (42.0)
3	536 (52.2)
Specialty	
Gynecological endocrinology	77 (7.5)
Gynecology	465 (45.3)
Obstetrics	79 (7.7)
Obstetrics and gynecology	406 (39.5)
History of clinical confirmed diseases	
Hyperlipidemia	66 (6.4)
Cardiovascular diseases	36 (3.5)
Diabetes mellitus	16 (1.6)
Low bone density/osteoporosis	29 (2.8)
Hyperthyroidism/hypothyroidism	5 (0.6)
Menstrual status	
Amenorrhea (menopause and total hysterectomy)	197 (19.1)
Oligomenorrhea	72 (7)
Moderate menstrual flow	648 (63.1)
Menorrhagia	110 (10.6)
Reasons for induced abortions	
Unreliable contraceptive method	317 (55.5)
Failure of reliable contraceptive method	108 (18.9)
Unintended pregnancy in spite of reliable contraceptive method	64 (11.2)
Use of emergency contraception	24 (4.2)
Disease	5 (0.9)
Medications used during pregnancy	7 (1.2)
Suspending embryo growth	3 (0.5)
Abnormal development of fetus	3 (0.5)

### Choice of contraceptive methods

A total of 367 OB/GYNs who were sexually active did not use any contraception because of amenorrhea (*n* = 197; 53.67%) or a plan to conceive (*n* = 170; 46.32%). A total of 516 parous OB/GYNs who had completed their families and 120 nulliparous OB/GYNs who did not plan to conceive at present reported using contraceptive methods. The contraceptive methods used are listed in Table 2. IUDs were the most commonly used method in parous women compared with nulliparous women (42.6% vs 1.7%; *p* < 0.0001), followed by condom plus rhythm method (*n* = 150, 24.2% vs 20.8%), condom (used every time, *n* = 54, 10.5%), and COCs (*n* = 26, 4.4% vs 2.5%). On the contrary, the use of rhythm method only (*n* = 75, 48.3% vs 3.3%; *p* < 0.0001) was high among nulliparous women compared with parous women, followed by the use of condoms alone (*n* = 77, 19.2% vs 10.5%; *p* = 0.008). Although IUDs were commonly used in parous women, LNGIUS use remained as low as 1 and 0.8% in parous and nulliparous women, respectively.

**Table 2:** Use of contraceptive methods among OB/GYNs or by their partners

Method	Nulliparous, *n* (%) *N* = 120	Parous, *n* (%) *N* = 516	*p* value
Mean age, years	27.7	40.0	< 0.0001
IUD	2 (1.7)	220 (42.6)	< 0.0001
LNG-IUS	1 (0.8)	5 (1.0)	1.000
Condoms	23 (19.2)	54 (10.5)	0.008
Condom + rhythm	25 (20.8)	125 (24.2)	0.431
Condom + contraception for external use (spermicide)	7 (5.8)	27 (5.3)	0.792
COC	3 (2.5)	23 (4.4)	0. 392
Long-acting oral contraceptives	0 (0)	3 (0.627)	–
Subdermal implant	0 (0)	2 (0.4)	–
Female sterilization	0 (0)	2 (0.4)	–
Vasectomy	0 (0)	1 (0.01)	–
Coitus interruptus	1 (0.8)	37 (7.1)	0.005
Rhythm	58 (48.3)	17 (3.3)	< 0.0001

### Benefits and side effects of COCs and LNGIUS

In response to the question of additional benefits, most OB/GYNs reported that COCs are able to relieve dysmenorrhea and premenstrual tension syndrome (73.8%, *n* = 758); treat polycystic ovary syndrome (68.1%; *n* = 699); cure acne and improve sebum secretion (67.95%, *n* = 697); decrease menstrual flow and improve anemia (64%, *n* = 657); and reduce the risks of endometriosis, ovarian cancer, and endometrial carcinoma (61.5%, *n* = 632). Only 32.8, 28.8, and 28.5% of the OB/GYNs reported that COCs are able to reduce the incidence rate of pelvic inflammation, cure cutaneous pigmentation, and mitigate melancholia, respectively. Four OB/GYNs were unclear about the additional benefits of COCs. Further details regarding the knowledge of OB/GYNs on clinical application of COCs and side effects are shown in Table 3.

**Table 3:** OB/GYNs’ knowledge and perceptions of contraceptives clinical application and side effects

Questions	Yes, *n* (%)	No, *n* (%)	Don’t Know, *n* (%)
• Gestational trophoblastic patients may take low dose of COC	210 (20.4)	500 (48.7)	317 (30.9)
• Should consider pregnancy 3–6 months after stopping the intake of COC	252 (24.5)	681 (66.3)	94 (9.2)
• Taking COC for long term is harmful to health; however, interrupted intake is recommended	193 (18.8)	706 (68.7)	128 (12.5)
• Taking COC may reduce the fertility of women	77 (7.5)	822 (80.0)	128 (12.5)
• Taking COC for a long term may cause amenorrhea and premature ovarian failure	109 (10.6)	779 (75.9)	139 (13.5)
• Taking COC may increase the risk of thrombi formation	675 (65.7)	215 (20.9)	137 (13.3)
• Taking COC may increase the risk of breast cancer	381 (37.1)	466 (45.4)	180 (17.5)
• Taking COC may cause weight gain	511 (49.8)	376 (36.6)	140 (13.6)

Similarly, majority of the OB/GYNs reported that LNGIUS had additional benefits over a copper IUD in terms of reducing menstrual blood flow and improving menorrhagia and anemia (81.0%, *n* = 831); relieving dysmenorrhea (77.9%, *n* = 800); protecting uterine endometrium, preventing endometrial hyperplasia, and endometrial carcinoma (77.0%, *n* = 791); curing or preventing endometriosis (59.9%, *n* = 615); reducing pelvic inflammatory disease risk (35.1%, *n* = 360); and reducing the risk of ectopic pregnancy (0.2%, *n* = 2).

## Discussion

This survey was the first of its kind to investigate the contraceptive choice of Chinese OB/GYNs. To understand the factors that influence the physician’s recommendation of contraceptive methods, it is essential to understand the pattern of contraceptive use. A survey on perspectives of HCPs on contraceptive use revealed that misconceptions and lack of knowledge on the long-term effects of contraceptives may have influenced contraceptive counseling and provision [10]. As OB/GYNs play a major role in counseling and recommendation of contraceptive methods [11], their perceptions and knowledge are of immense importance to meet the unmet needs for contraception and to develop strategies for reducing unintended pregnancies and abortions [12]. The rate of induced abortion because of unintended pregnancy among the OB/GYNs in the present study was as high as 56.3%, which was largely due to the use of unreliable contraceptive methods such as rhythm method and lactation amenorrhea. This was similar to previous studies in Chinese women who reported an absence or poor use of contraceptives as the main cause of unplanned pregnancies, followed by failure of withdrawal, timing, and emergency contraceptive methods [13–15]. In this study, among the OB/GYNs who had completed their families, use of the IUD was high. The proportion of female sterilization (0.2%) and vasectomy (0.1%) were both low, indicating that OB/GYNs preferred reversible methods. Except for the IUD, contraceptive methods of choice were short-acting. Among the 120 nulliparous OB/GYNs, more than half chose unreliable methods. This suggests that OB/GYNs preferred rhythm method and had insufficient knowledge about the high failure rate of condom and rhythm method, which was the leading cause of induced abortion due to unintended pregnancy among female OB/GYNs.

Studies have found that healthcare providers, family physicians, and OB/GYNs have inadequate knowledge about contraceptive methods and outdated information about the safety of IUDs [5, 6, 16, 17]. The authors agree with these reports, as the use of IUDs was low among nulliparous OB/GYNs in the present survey. It may be plausible that the young unmarried OB/GYNs were more concerned about the perceived infertility associated with IUDs as they were yet to complete their families unlike the parous OB/GYNs who completed their families. These misconceptions may result from information indicating an inappropriateness of IUDs in nulliparous women as these women have smaller uterus that might increase the expulsion rate and increase the pain with insertion [18]. Similar to this study, previous studies from Western countries have reported a lack of awareness and misconceptions among HCPs regarding the unsuitability of IUD in certain groups of women, levying a substantial impact on the IUD use [19]. In addition, LNGIUS use in this study was low (0.5%), despite its availability in the Chinese market since 2000 and high satisfaction rates among Chinese women [20]. The reason for the low prevalence may be the high price, which is not covered under the health insurance schemes. However, OB/GYNs demonstrated knowledge of non-contraceptive therapeutic actions and benefits of LNGIUS.

In this study, a high proportion of female OB/GYNs had misconceptions about the clinical application of COCs, such as reduced fertility, harmful long-term effects, amenorrhea and premature ovarian failure, increased risk of breast cancer, and weight gain. With various continuing education programs in the recent years, knowledge of OB/GYNs on reproductive safety of COCs has improved; 66.3% of the respondents reported that it is good to get pregnant after discontinuing COCs. However, concern about the side effects of COCs may lead to decreased use of COCs among OB/GYNs. In addition, this concern may influence COC use among the general population. Another reason for low COC use might be the preference toward long-acting contraceptive methods in the family planning policy. This perception is in concordance with previous findings that women were unlikely to prefer everyday pills because of the concern of side effects and hazard to overall health [21–23]. Although COCs are not the preferred choice of contraception among female OB/GYNs in this study, they are highly recognized as rendering benefits other than contraception, such as treating dysmenorrhea, relieving premenstrual tension, treating acne and improving sebum secretion, reducing menstrual flow and improving anemia, treating endometriosis, and decreasing the risks of ovarian cancer and/or endometrial carcinoma.

Overall, we observed that most OB/GYNs preferred long-acting, reversible, cost-effective contraceptives with least side effects. According to previous studies, these factors influenced HCP recommendation of contraceptives [4].

This study has several limitations. The main limitation was that the questionnaire was exclusively designed for OB/GYNs and was not intended to evaluate the contraceptive recommendation given by them to their patients. In addition, the survey was conducted in 2013 and involved only OB/GYNs from urban areas, which may lead to selection bias and non-generalizability of the findings to OB/GYNs in rural areas of China. Also, the questionnaire was exhaustive and some respondents found it too cumbersome to complete. The survey findings reveal the lacunae in the knowledge and perceptions of the OB/GYNs on contraceptive choice and use. This warrants caution and a need to provide effective education about the safe and effective use of contraceptive methods to OB/GYNs to reduce the rate of unintended pregnancies in the rest of the population in China.

## Conclusions

In summary, the high rate of induced abortion among OB/GYNs in urban China may be influenced by the use of unreliable contraceptive methods, which is due to misconceptions regarding reliable methods such as COCs and LNGIUS. In addition, the use of IUDs was dependent on the perceptions based on parity of the individual OB/GYN, wherein the nulliparous OB/GYNs were unlikely to use IUDs because of perceived infertility in the future. Hence, continuous education about the safe and effective use of contraceptive methods should be strengthened among OB/GYNs to overcome the misconceptions and fear of side effects to ensure the unbiased recommendation of contraceptive methods to the general public.

## Abbreviations

COC: Combined oral contraceptive
HCP: Healthcare professional
IUD: Intrauterine device
LNGIUS: Levonorgestrel intrauterine system
OB/GYN: Obstetrician/Gynecologist
